# Factors Affecting Two Types of Memory Specificity: Particularization of Episodes and Details

**DOI:** 10.1371/journal.pone.0166469

**Published:** 2016-11-16

**Authors:** Rebecca M. Willén, Pär Anders Granhag, Leif A. Strömwall

**Affiliations:** 1 IGDORE, The Globally Distributed Institute for Open Research and Education; 2 Department of Psychology, University of Gothenburg, Gothenburg, Sweden; 3 Norwegian Police University College, Oslo, Norway; University of Akron, UNITED STATES

## Abstract

Memory for repeated events is relevant to legal investigations about repeated occurrences. We investigated how two measures of specificity (number of events referred to and amount of detail reported about the events) were influenced by interviewees’ age, number of experienced events, interviewer, perceived unpleasantness, and memory rehearsal. Transcribed narratives consisting of over 40.000 utterances from 95 dental patients, and the corresponding dental records, were studied. Amount of detail was measured by categorizing the utterances as generic, specific, or specific-extended. We found that the two measures were affected differently by all five factors. For instance, number of experienced events positively influenced number of referred events but had no effect on amount of detail provided about the events. We make suggestions for future research and encourage reanalysis of the present data set and reuse of the material.

## Introduction

How many times have you visited a dental clinic? Maybe about once a year since you were a child? That would be about… 20 times? 40? Now try to recall individual episodes of your dental visits. Maybe you remember the last visit you made. What about the first visit you made? Do you perhaps remember visits that were somehow different from the others? If you do give it some time, you might end up recalling about a handful of individual dental visits that you have made during the past ten years, even if you in fact have made several dozens of visits during those ten years [1]. This is one way to quantify your memory for the dental visits you have made. A different way is to look closer at the words you use when describing the dental visits you recall. To which extent do you describe visits in a very precise/specific way (e.g., “that visit took place in January, just before my birthday”)? To which extent do you fail to remember such specific information and instead satisfy with a more general description (e.g., “my visits usually take place during the winter”)? This type of calculation may show that you can provide somewhere between 100 and 300 specific utterances overall about the visits you have made during the past ten years [1]. Thus, you may be able to recall about 6 individual episodes (e.g., 6 dental visits) and about 100–300 specific information units about your dental visits.

Differentiating between these two measures of memory specificity are relevant when it concerns recollection of events that have occurred repeatedly. Examples of events that typically are experienced repeatedly are restaurant visits [2], health visits [3], dental visits [1], and family gatherings [4]. It is also common for criminal events such as intimate partner violence [5]and child sexual abuse [6] to occur repeatedly.

Memories about similar events that have been experienced repeatedly tend to be less complete [7, 8] and more general [9] than memories about events that occurred only once. Although details that are consistent across events are likely to be remembered well [10], the individual events can become less distinct with recurrence which in turn makes remembrance of individual episodes more difficult [11] (the distinctiveness principle, [12]). In addition, recurrence increases the risk of ascribing an otherwise correct detail to the wrong event [13, 14]. These problems are rarely an important issue in daily life because we have no real reason to accurately remember everything we experience. However, in for example criminal investigations of repeated sexual abuse or intimate partner violence it is important that the plaintiff does not only describe details about the events in general, but also (1) provides specific information about individual episodes and (2) can refer to a number of episodes (e.g., more than two; [15]). Hence, there is a fast growing body of psychological research on how to facilitate recollection of repeated events in legal interviews. Although the present study concerns adults' memories for repeated events, most research on the topic has so far concerned children's memories. A recent review [16] summarizes mnemonics suggested to be of use in police interviews with children. Recommended techniques to increase the amount of detail recalled include the use of open-ended questions, asking for the gist before asking about specific episodes, and allowing the alleged victim to practice memory specificity before interviewing about the target events. In addition, recollection of individual events may be facilitated if the interviewer employs specific language (e.g., *“What else happened*, *the time she was at the shops*?*”*; p. 8), adopts labels and the witness' own words for events, and follows up on details mentioned by the interviewee by asking whether it occurred once or more than one time [16]. Additional techniques were suggested. For instance, asking about differences between the events may generate more details [16], and the constructing of a time-line may generate temporal information about the individual events [17]. Although there are only a handful of studies that have investigated particularization of adults' memories, the recommended mnemonics for use with children are typically the same for adults. Components from the Cognitive Interview (CI; [18]), for example open-ended questions, seem to facilitate particularization of individual events [7] and details about the events [4, 19]. Two other studies [3, 8] tested the effect of CI-components in combination with additional memory enhancing components such as constructing a time-line, asking about the first event and the most recent event, and whether anything unusual occurred in connection to any event. This package of mnemonics successfully increased recollection of individual events, while amount of detail never was measured. A similar package of techniques, although less extensive, was employed by [4], basically replicating the results by Means et al. [3, 8]. Previous research on adults had never tested individual mnemonics, which Willén and colleagues [1] made an effort to change when testing the usefulness of one of the mnemonics (context-specific/derived cues) successfully employed by Leins et al. [4]. The study by Willén et al. [1] was the first to make an explicit distinction between recollection of individual events and recollection of amount of detail about the events. The results showed different effects of context-specific cues on memory for individual events and memory for details about the events. This unexpected difference inspired the current work.

### The present study

The present study extended the work by Willén et al. [1] by further investigating the two measures of memory specificity mentioned above: number of individual events recalled and amount of detail provided about the events. The study was exploratory, aiming at providing new understanding about employed measures of memory specificity in research on repeated events. More specifically, we tested how these two measures were affected by five factors that the existing data set allowed us to explore: 1) the age of the (adult) interviewee, 2) the number of events experienced, 3) who had conducted the interview, 4) how unpleasant the interviewee found this type of events (dental visits) to be, and 5) the extent to which the interviewee had rehearsed the memories by thinking or talking about them. Hence, we investigated the effects–if any–these factors had on the number of individual events recalled and the amount of detail provided about the events.

The rationale for testing these particular factors was that they all have implications for applied settings, especially legal ones, and could quite easily be controlled (e.g., interviewer) or investigated (e.g., the age of the witness and the number of events reported to have occurred).

It should be noted that the effects of unpleasantness, rehearsal and interviewer on number of events and details about the events was partly investigated in Willén et al. [1]. However, two categories of details (specific and specific-extended memories; see the Methods section) were merged in the previous study. In the present study these two categories are analysed separately, to make the analyses more comprehensive but also comparable to the other analyses presented in the current paper. In addition, in the previous study we did not explore the proportions and the generic memories were thereby disregarded.

## Method

### Participants and procedure

Participants were 95 adult dental care patients (71 women). Their mean age was 43.33 years (*SD* = 15.17). Initially there were 99 participants but four were excluded: two because they did not bring their dental records and two due to interviewer mistakes when interviewing. Participation was voluntary and each respondent received a gift card valid at stores and restaurants, worth about 28 Euro.

All participants brought their dental records to the data collection session in envelopes sealed by the personnel at the dental clinics to make sure our respondents had not seen the records prior to participating. A written informed consent form regarding the use of dental records for research purposes was collected from each participant.

Participants were interviewed once (a second interview was conducted after this—described in Willén et al. [1]—but it is excluded here since it is not relevant to the current study) and asked to recall dental visits they had made during the past ten years (2002 to 2012). They were instructed to tell about all visits they could recall having made during this period, no matter which clinic it concerned or whether we had access to the dental records from that particular clinic.

Prior to the interview, participants were left alone for 5 minutes to prepare by thinking back on the dental visits they had made. The interview was conducted by one of four trained interviewers, who each met with between 19 and 31 respondents. Before the interview started, participants were instructed to avoid guessing, and it was emphasised that they could ask questions at any time during the interview.

The interviewers followed a structured interview protocol. The first two questions were: *“In total*, *how many dental visits have you made during the past 10 years*? *[…] How many of those were at the clinic(s) from which you have dental records*?*”* Participants were then asked to choose any one of the visits and tell everything they remembered about it (free recall). The interviewer continued with specific questions until the respondent claimed to not recall anything more. The procedure with free recall and specific questions was then repeated for each individual visit that the participant could refer to. The interview usually took about 30 minutes but sometimes up to an hour.

Afterwards participants answered a questionnaire. Apart from questions about sex and age the respondents were also asked questions about, for example, how unpleasant they found dental visits to be and how much they had talked and thought about their dental visits (responses were given on rating scales ranging from 1 to 7).

For full details on the procedure, see [20].

### Coding and data preparation

All interviews were transcribed verbatim.

#### Individual events

One research assistant coded all 95 statements for number of referred visits and subjective (estimated) number of experienced events, and the dental records for objective number of experienced events. Independently of the first coder, another assistant conducted the same coding for 20 randomly selected statements with dental records. Intraclass correlations were calculated, showing excellent interrater agreements of .96, 95% CI [.91, .99] for referred events, 1.00 for subjective number of experienced events, and .997, 95% CI [.994, .999] for objective number of experienced events.

Subjective number of events was recoded for 34 participants before investigating the accuracy of respondents' estimations. Recoding was necessary because these respondents had made visits to clinics from which we had no dental records. To avoid data exclusions we instead used their estimates of how many times they had been to certain clinics (from which we did have their dental records). In two cases we also had to recode the objective number of visits since we had several dental records from the participant but not self-reported estimates for each clinic.

#### Details about the events

The transcripts were broken down into short utterances similar to the procedure employed in the study by Orbach, Lamb, Sternberg, Williams, and Dawud-Noursi [21]. Two assistants shared the workload and followed a written manual. Their work resulted in more than 40.000 utterances. A computer program was created to automatically transfer the utterances from a Word file to an Excel file, and the latter was prepared to make the coding procedure more intuitive for the assistants. In the Excel file, each utterance was categorised into one of five categories that are commonly employed when measuring overgeneral memory (Autobiographical Memory Test, AMT; [22]): *Interviewer*–questions or responses by the interviewer; *Error*–not related to the dental visits or not containing any (new) information (e.g., “I don’t know” and repetitions); *Generic*–summaries of how something usually or typically occurs (e.g., “because I’m often very dry in my mouth when being stressed”); *Specific*–a memory of something particular which lasted within 1 day (e.g., “they had a trainee there during that visit”); *Specific-extended*–a memory of something particular which lasted more than 1 day (e.g., “During that period I had a lot of acne”). One assistant categorised all statements, and a second assistant categorised 21% of the statements. The interviewer category was excluded in the agreement calculations. The interrater reliability was initially not impressive (.58, unweighted Cohen's kappa). Therefore, a training session was held with both coders where after an acceptable agreement was achieved .66, 95% CI [.65, .67]. The main agreement issues concerned when to categorize an utterance as Error and how to differentiate Specific memories from Specific-extended.

In addition, the narratives were compared to the dental records and coded for verification. The full results are presented in Willén et al. [1]. In short, specific memories could be verified almost twice as often as the generic memories, and the incidence of verified specific-extended memories was in between the other two types of memories [1].

### Shared data and materials

All materials and codes used for the purpose of this study is available on the Open Science Framework [23]. The data, supplemented with a codebook, is also available online [20]. Hence, we welcome reuse of the data and material, as well as refining of the material.

### Statistical analyses

No additional factors than those we report (i.e., age, interviewer, number of experienced events, unpleasantness, and rehearsal) were investigated, and no additional analyses to test them were conducted than those we explicitly report. However, comments on an earlier version of the manuscript suggested that we control for interview length in all our analyses. All analyses were then re-run accordingly and the results were indeed quite different (e.g., interviewer had an effect on memory for individual events but no longer any effect on amount of detail). However, in our final draft we only include the original analyses, and have thus refrained from controlling for interview length. This decision was made because of two reasons. (1) In part, interview length is already controlled for in the regression analyses because interviewer is included as a control variable. (2) When controlling for interview length, the analyses of interviewer effects lost their meaning: Our interpretation is that one of the interviewers held longer interviews because the participants told her more details. The opposite relation (i.e., that longer interviews provide more space for the interviewees to recall more) is possible, but we find it less likely. All outputs from the analyses controlling for interview length are available online [24].

Two-tailed tests were employed and alpha level was set at .05.

All the regression analyses were hierarchical with 5 steps. The order of the predictors was: (1) control variables, (2) demographic variables, (3) chronological order based on expected influence on the outcome (largest influence first).

For 61 respondents the objective number of events was equal to all visits made during the past ten years since they had not been to any other clinics. However, for 34 respondents the objective numbers were smaller than the total number of experienced events (which is unknown to us), because they had made visits to clinics from which we had no dental records. We therefore conducted separate analyses of objective and subjective numbers of visits, to make sure that the analyses were as comprehensive as possible.

## Results

### Preliminary and descriptive analyses

The mean length of the interview was 30.23 minutes (*SD* = 16.04). A one-way ANOVA showed significant differences in interview length between interviewers, *F*(3,91) = 9.33, *p* < .001, η^2^ = .24. A post hoc test (Bonferroni) showed that one interviewer (*M* = 45.01, *SD* = 19.19) held significantly longer interviews than the other three interviewers (*M* = 26.16, *SD* = 12.63; *M* = 24.74, *SD* = 11.90; *M* = 28.53, *SD* = 13.56), all *p*’s ≤ .003. This large difference between interviewers was unwanted, but it enabled us to study the effect of interviewer on the two measures of specificity.

A paired samples *t*-test comparing the recoded variables of subjective (estimated) number of visits (*M* = 14.74, *SD* = 9.26) and objective number of visits (*M* = 19.55, *SD* = 14.91) revealed that the respondents underestimated how many dental visits they had made, *t*(34) = -4.88, *p* < .001, *r* = .45. After excluding likely rounding errors (i.e., estimations that were wrong with < 5 events) we found that five respondents overestimated the number of visits (ranging from 5 to 16 visits), while 30 participants underestimated the number with on average 15 visits (ranging from 5 to 45).

The assumption of no multicollinearity was not violated in any of the regression analyses. Utterances categorized as error (about 22% of the utterances; [1]) were excluded, where after mean proportions of the type of memories were calculated. The sum (i.e., 1) consisted of generic (*M* = 0.07, *SD* = 0.05), specific-extended (*M* = 0.19, *SD* = 0.09), and specific memories (*M* = 0.74, *SD* = 0.11). The proportions formed the basis for all regression analyses on type of memory (but raw numbers were used in the MANOVA testing interviewer effects), and interviewer was controlled for since we tested the effects of interviewer separately.

### Age

A hierarchical regression analysis showed no effect of age on the number of referred events, *B* = -0.02, *p* = .52, 95% CI [-0.06, 0.03]. However, the number of experienced events increased with age [1]. We therefore conducted a separate hierarchical regression analysis where number of experienced events and interviewer were controlled for. Age did not predict number of referred events, although a slight decrease of referred events with age could be seen, *B* = -0.04, *p* = .10, 95% CI [-0.10, 0.01]. In turn, three hierarchical regression analyses showed that increased age generated small but statistically significant changes in all three types of memories: an increase of generic memories, *B* = 0.002, *p* < .001, 95% CI [0.001, 0.002], a decrease of specific memories, *B* = -0.003, *p* < .001, 95% CI [-0.004, -0.002], and an increase in number of specific-extended memories, *B* = 0.002, *p* = .01, 95% CI [0.00, 0.003]. Thus, as summarized in Table 1, age affected only one of the two specificity measures.

**Table 1:** The influence of the five different factors on two measures of recollection.

Factor	Referred events	Referred details
Age	No	Yes
Number of events experienced	Yes	No
Interviewer	No	Yes
Unpleasantness	Yes	No
Rehearsal by talking	Yes	No
Rehearsal by thinking	No	No

### Number of experienced events

As reported in Table 2, there was a small but statistically significant increase of referred events as the objective number of experienced events increased, *B* = 0.06, *p* = .03, 95% CI [0.00, 0.11]. This result was stronger when subjective number of experienced events was employed instead of the objective numbers, *B* = 0.13, *p* < .001, 95% CI [0.06, 0.20]. There were however no statistically significant changes with regard to type of memory (see Table 2): generic memories, *B* = -0.00, *p* = .97, 95% CI [-0.00, 0.00]; specific memories, *B* = 0.00, *p* = .52, 95% CI [-0.00, 0.00]; specific-extended memories, *B* = -0.00, *p* = .47, 95% CI [-0.00, 0.00]. The results were in the same directions with subjective number of visits as the predictor: generic memories, *B* = -0.00, *p* = .95, 95% CI [-0.00, 0.00]; specific memories, *B* = 0.00, *p* = .12, 95% CI [0.00, 0.00]; specific-extended memories, *B* = -0.00, *p* = .08, 95% CI [-0.00, 0.00]. Hence, type of memory seemed unaffected by number of experienced events.

**Table 2:** Hierarchical multiple regression analyses predicting number of referred events and type of memory (amount of detail) from age, number of experienced events, unpleasantness and rehearsal.

				Type of memory					
		Events		Generic		Specific		Spec.-ext.	
Predictor		Δ*R*^2^	β	Δ*R*^2^	β	Δ*R*^2^	β	Δ*R*^2^	β
Step 1		.03		.06		.06		.09^*^	
	Control variable^*a*^								
Step 2		.00		.19^***^		.18^***^		.07^**^	
	Age		-.07		.44^***^		-.44^***^		.26^**^
Step 3		.05^*^		.00		.00		.01	
	Experienced events^*b*^		.26^*^		-.00		.07		-.08
Step 4		.06^*^		.01		.01		.00	
	Unpleasantness^*c*^		.26^*^		-.12		.11		-.06
Step 5		.11^**^		.07^*^		.01		.00	
	Thought^*c*^		.00		-.18		.03		.07
	Talked^*c*^		.35^**^		-.16		.09		-.01
Total *R*^2^		.25^**^		.34^*^		.26		.16	
*n*		95		95		95		95	

*Notes*.

^*a*^ Control variables include the dummy coded variable interviewer.

^*b*^ Only the results from objective numbers are presented here.

^*c*^ These results were partly reported in Willén et al. [1], see *“The present study”*.

^*^ *p* ≤ .05

^**^ *p* ≤ .01

^***^ *p* < .001.

### Interviewer effects

A one-way ANOVA showed no statistically significant effect of interviewer on the number of referred visits, *F*(3,91) = 1.06, *p* = .37, η^2^ = .03. However, a one-way MANOVA with type of memory (generic, specific-extended, and specific in raw numbers, i.e., not proportions) as dependent variables showed a significant effect of interviewer, Wilks’ *F* (9, 216.75) = 3.98, *p* < .001, partial η^2^ = .12. All univariate tests were statistically significant and showed medium sized effects [25]: generic, *F* (3, 91) = 7.48, *p* < .001, partial η^2^ = .20; specific-extended, *F* (3, 91) = 8.25, *p* < .001, partial η^2^ = .21; specific, *F* (3, 91) = 5.90, *p* = .001, partial η^2^ = .16. Post hoc tests revealed that the interviewer who held the longest interviews (as reported under *Preliminary analyses*) extracted more information than the other three interviewers, and this finding held for all types of information: generic (Games-Howell [due to unequal variances]; *p*’s between .03 and .08), specific-extended (Games-Howell; all *p*’s except one were < .02), and specific (Bonferroni; all *p*’s ≤ .01). Thus, as summarised in Table 1, interviewer had a significant effect on one of the specificity measures but not the other.

### Unpleasantness

As previously reported [1], higher levels of unpleasantness were associated with a statistically significant increase of referred events, *B* = 0.41, *p* = .01, 95% CI [0.08, 0.73]. Unpleasantness did however not seem to affect the types of memories reported: generic memories, *B* = -0.00, *p* = .21, 95% CI [-0.01, 0.00]; specific memories, *B* = 0.01, *p* = .27, 95% CI [-0.00, 0.02]; specific-extended memories, *B* = -0.00, *p* = .59, 95% CI [-0.01, 0.01].

### Rehearsal

As reported in Willén et al. [1], the extent to which the respondents had *talked* about their dental visits positively influenced how many visits they could refer to, *B* = 0.82, *p* = .003, 95% CI [0.29, 1.36], while it did not matter how much they had *thought* about their visits, *B* = 0.01, *p* = .98, 95% CI [-0.45, 0.46]. Higher levels of rehearsal were associated with a minor decrease in generic memories: thinking; *B* = -0.01, *p* = .10, 95% CI [-0.01, 0.001]; talking; *B* = -0.01, *p* = .14, 95% CI [-0.01, 0.002]. There were no statistically significant differences with regard to specific memories (thinking; *B* = 0.00, *p* = .78, 95% CI [-0.01, 0.02]; talking; *B* = 0.01, *p* = .46, 95% CI [-0.01, 0.02]) or specific-extended memories (thinking; *B* = 0.00, *p* = .56, 95% CI [-0.01, 0.02]; talking; *B* = 0.00, *p* = .96, 95% CI [-0.02, 0.02]).

## Discussion

We investigated how two measures of specificity, namely recall of individual events and recall of details about the events, were influenced by five factors that can be controlled or investigated for the purpose of interviewing a witness about repeated events. We found that the two outcome measures were affected differently for the five factors. Recollections of individual events were positively affected by an increase in number of experienced events and high levels of unpleasantness. Memory performance for individual events was also enhanced if the respondents had talked much about the events to someone (however, thinking about the events showed no effect on neither measure). In contrast, the amount of detail about the events was not affected by any of these three factors, but there was a decrease of details–but not events–with age. Interviewer had an effect on amount of detail, but not on recollection of individual events.

### Main findings

The one interviewer that held lengthy interviews generated more detailed accounts than the three other interviewers. As implicitly suggested by Leins et al. [4], a lengthy and thorough interview might not be important when trying to extract as many individual events as possible, although a thorough interview may be necessary for extracting details about the individual events. For extracting individual events it might prove more helpful to employ particular cueing methods such as a timeline (e.g., [17]) and context-specific cues [1]. It is thus important to conduct more studies on specialized techniques, which so far has been rare in research on adults’ memories for repeated events (see [1], for an exception). However, an alternative explanation for why the one interviewer generated more details but no additional events is that interviewees possibly had no energy and motivation to continue talking about the events. Through introspection most of us can probably agree that there certainly is a limit to the amount of talking we are willing to do about certain events, whether they are happy, traumatic or neutral. Short and long breaks may provide new energy and information, but the recharging effect is likely to decline as the interviews continue or are repeated. Thus, more generic information [26] or less information with repeated interviews may not only be a result of memory exhaustion, but of failed motivation. For a practitioner, it may therefore not only be a question of choosing useful memory enhancing-techniques, but also an investigate decision regarding which type of information that is of highest priority in a particular case, for example whether the witness is preferred to describe individual events or details about the events.

The number of experienced events did not affect the amount of detail recalled. This result suggests that one should not necessarily expect a more detailed account due to a reportedly large number of experienced events. In contrast, increases in number of experienced events had a positive effect on recollection of individual events. The *B*-value showed that for a unit increase in number of experienced events, the number of referred events increased with between 0.06 (objective numbers) and 0.13 (subjective numbers). That is, for every 10 visits experienced the respondents could refer to between 0.5 and 1.5 visits. In applied legal settings this might mean that a witness’ own estimate of how many times s/he has experienced the event may be useful for making a rough prediction of how many events the witness should be able to mention and provide at least some details about. Such a rough prediction could for instance be that someone who believes s/he has experienced 40 repeated events can be expected to mention about 6 events. However, it is crucial to note that our data showed a large variance with regard to number of referred visits. Some respondents had made 30–40 dental visits but could not refer to more than two or three of them. It is up to future research to further explore whether it might be possible to say with any certainty how many events that can be expected of an adult to recall, and whether our null result regarding the amount of detail will replicate.

Higher ratings of dental visits as generally unpleasant were associated with increased recollection of individual events, but without the corresponding effect on amount of detail recalled about the events. Arguably, people who feel uncomfortable with dental visits worry more than others before the visits take place, and consequently this particular type of event (i.e. dental visits) become more distinct compared to similar types of events (e.g. other health visits or 'must do' errands). Such a distinctiveness may enhance memory for the visit taking place [12]. Thus, more individual events can be particularized, but without a coincident increase in amount of detail.

Rehearsal of repeated events can facilitate recollection [27]. Our results supported this finding for the remembrance of individual episodes, but not for remembrance of details about the events. When assessing credibility and reliability in legal investigations it might be useful to consider the extent to which a plaintiff or witness has talked about the events to someone. Evidently, talking can facilitate recollection.

In line with previous research (e.g., [28]) showing that older adults’ recollections tend to be generic, we found an increase of generic memories with age. However, the corresponding decrease of referred events was not statistically significant. It is possible that memory for events is less affected by adults' age than memory for details about the events. It should be noted though that a small decrease of referred events with age was shown and a replication with a larger sample may show a more significant decrease.

Finally, a side finding is worth mentioning. Plaintiffs are commonly asked to provide an estimate of the number of times they were abused [29] and it is important for practitioners to know how to evaluate the answer given. Our study adds to previous studies (e.g., [30, 31]) suggesting that underestimation is common. Thus, if the events truly occurred and if the number of events is large, the estimate is most likely on the low side.

### Limitations

We find two limitations of the present study to be the most important. First, we investigated the influence of five factors on two different measures of specificity. The choice of factors was made after the data collection and was primarily based on what possibilities the collected data offered. This calls for cautiousness when drawing conclusions from the results and it is up to future research to investigate whether they are replicated.

Second, our results are based on all referred events and details mentioned by the respondents. We could have conducted analyses on verified events and details only. However, we would then had to exclude data and since the purpose of the present study was exploratory we found it more important to include all data rather than having it verified. Information about the extent to which the data was verified by the dental records is found in the study by Willén et al. [1].

## Conclusions

The present study points to the importance of distinguishing between two types of specificity when it regards memory for repeated events: number of individual events recalled and amount of detail provided about the events. Our results suggest that particular mnemonics (e.g., type of interview or questions) and other factors (e.g., number of experienced events or trauma) are likely to affect these two measures differently. It is thus possible that techniques for particularization of repeated events can become more effective if specialized towards generating either individual events or details about the events. Additionally, we suggest that failed motivation is likely to strike the interviewee sooner or later regardless of situation and topic. It may therefore not only be a question of specializing techniques, but also an investigative decision on behalf of the practitioners regarding which type of information that is of highest priority in a particular case.
